# At the intersection of non-coding transcription, DNA repair, chromatin structure, and cellular senescence

**DOI:** 10.3389/fgene.2013.00136

**Published:** 2013-07-26

**Authors:** Ryosuke Ohsawa, Ja-Hwan Seol, Jessica K. Tyler

**Affiliations:** Department of Biochemistry and Molecular Biology, University of Texas MD Anderson Cancer CenterHouston, TX, USA

**Keywords:** non-coding RNA, chromatin, genomic instability, aging, senescence

## Abstract

It is well accepted that non-coding RNAs play a critical role in regulating gene expression. Recent paradigm-setting studies are now revealing that non-coding RNAs, other than microRNAs, also play intriguing roles in the maintenance of chromatin structure, in the DNA damage response, and in adult human stem cell aging. In this review, we will discuss the complex inter-dependent relationships among non-coding RNA transcription, maintenance of genomic stability, chromatin structure, and adult stem cell senescence. DNA damage-induced non-coding RNAs transcribed in the vicinity of the DNA break regulate recruitment of the DNA damage machinery and DNA repair efficiency. We will discuss the correlation between non-coding RNAs and DNA damage repair efficiency and the potential role of changing chromatin structures around double-strand break sites. On the other hand, induction of non-coding RNA transcription from the repetitive *Alu* elements occurs during human stem cell aging and hinders efficient DNA repair causing entry into senescence. We will discuss how this fine balance between transcription and genomic instability may be regulated by the dramatic changes to chromatin structure that accompany cellular senescence.

## INTRODUCTION

Cellular senescence is an irreversible cell cycle arrest caused by intrinsic or extrinsic stress, such as shortened telomeres, DNA damage, induced oncogenes, or chromatin perturbation. This phenotype was first observed by Hayflick (1965) using human diploid cells which could undergo a limited number of cell divisions in *in vitro* culture. Various signaling pathway and regulatory factors of senescence have been reported over the last four decades (Campisi and d’Adda di Fagagna, 2007). Cellular senescence contributes to the aging process (Campisi and d’Adda di Fagagna, 2007; Baker et al., 2011). Aging is highly complex and poorly understood. It is characterized by time-dependent degenerative changes caused by genetic (intrinsic) or environmental (extrinsic) stimuli. Although once thought to be an amalgamation of random detrimental events, the identification of mutations and diets that promote longevity has led to the realization that particular cellular pathways influence the aging process. The challenge now is to understand the mechanistic basis of how these pathways and metabolic states regulate aging.

Although numerous different pathways for aging have been proposed, it is striking that human premature aging syndromes are caused by defects in the maintenance of genomic stability (Coppede, 2012), indicating that genomic integrity plays a key role in determining lifespan. In agreement, the accumulation of DNA damage is a hallmark of aging and senescing cells (Sedelnikova et al., 2004), and the DNA damage signal emanating from shortened telomeres triggers cells to enter a state of senescence (d’Adda di Fagagna et al., 2003; Takai et al., 2003; Herbig et al., 2004). In turn, genomic stability is closely intertwined with the chromatin structure. The chromatin structure not only regulates the accessibility of DNA damaging agents to the genome, but also plays critical roles in the signaling of DNA lesions and their repair (see Papamichos-Chronakis and Peterson, 2013 for a very nice recent review). Given the critical role of chromatin in regulating genomic stability and gene expression, it is tempting to speculate that some of the changes in gene expression and genomic integrity that occur during aging may be caused by the global changes to the chromatin structure that accompany aging. These chromatin changes span from the global loss of heterochromatin in aging human cells (Howard, 1996; Villeponteau, 1997; Kitano and Imai, 1998; Tsurumi and Li, 2012) to the global reduction of histone levels during mitotic aging in yeast (Feser et al., 2010) and human fibroblasts (O’Sullivan et al., 2010). Here we will discuss how non-coding RNAs contribute to the relationships among chromatin structure, genomic integrity, and cellular senescence.

## NON-CODING RNA TRANSCRIPTION IS INDUCED BY DNA DAMAGE

DNA damage is induced by various genotoxic stresses from inside the cell or from the environment. Arguably the most dangerous type of DNA lesion is a double-strand break (DSB) because a single DSB can lead to the loss of a chromosome arm during mitosis if not repaired, while its inaccurate repair can lead to chromosomal translocations or mutations. DSBs are repaired by either homologous recombination (HR) which copies the identical undamaged DNA information usually from the sister chromatid, or non-homologous end joining (NHEJ) in which the two DNA ends are ligated back together (reviewed in Ciccia and Elledge, 2010). Following the generation of a DSB, the cell mediates a highly orchestrated series of events termed the DNA damage response (DDR; reviewed in Finn et al., 2012; Papamichos-Chronakis and Peterson, 2013). The function of the DDR is at least five fold: (i) to arrest the cell cycle until the DNA damage is repaired (checkpoint activation), (ii) to upregulate gene expression of the repair machinery, and ultimately (iii) to remodel and restore chromatin structure around damage site, (iv) to repair the DNA molecule *per se* by recruitment of the DNA repair machinery, (v) to trigger apoptosis if there is too much DNA damage to be repaired. At an early stage of the DDR phosphatidylinositol 3-kinase-like protein kinase (PIKKs) family [ATM (ataxia-telangiectasia mutated), ATR (ATM and Rad3-related), and DNA-PKcs (DNA-dependent protein kinase)] and MRN (Mre11–Rad50–Nbs1) complexes are recruited to the DNA breaks. The PIKKs phosphorylate downstream checkpoint proteins, such as Chk1 and Chk2, and the histone variant H2A.X that is present in the chromatin around the breaks. Phosphorylated Chk1 and Chk2 phosphorylate various effector proteins to arrest the cell cycle to allow time for DNA repair. Phosphorylated H2A.X (called γH2A.X) assists to recruit other downstream signaling molecules that facilitate the DDR. The SWI/SNF (switch/sucrose non-fermentable) complex (ATP-dependent chromatin remodeling factor) triggers chromatin relaxation after UV treatment. This chromatin relaxation is involved in late stages of DNA repair because it does not affect the recruitment of early DDR response factors but later DDR response factors, such as xeroderma pigmentosum complementation group G (XPG) and proliferating cell nuclear antigen (PCNA) to UV damage sites (Zhao et al., 2009). The proteins that mediate the DDR are conserved from yeast to human, meaning that our understanding of the DDR can be gained from studies in many different model systems.

**FIGURE 1:**
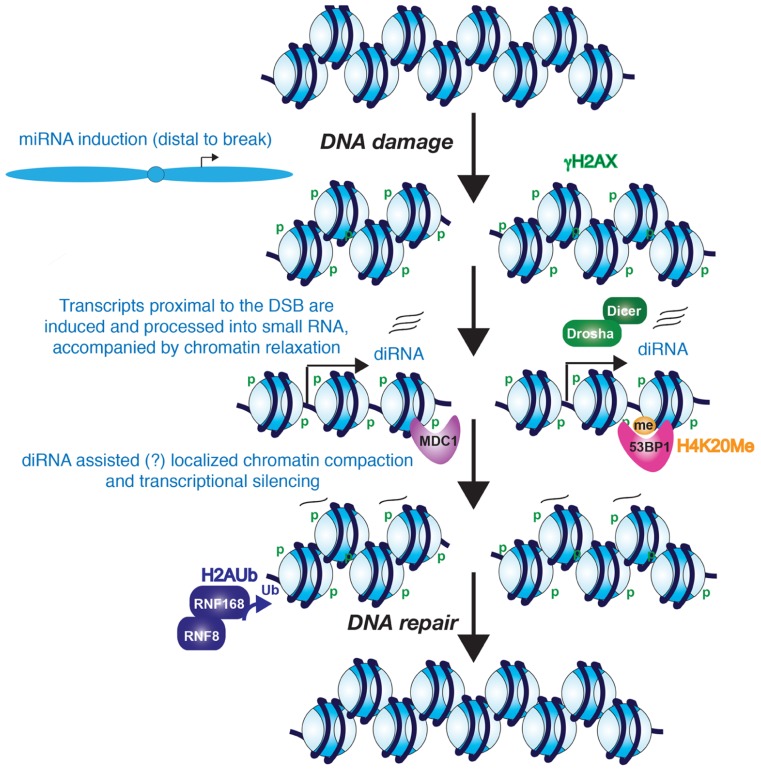
**Potential role for diRNAs in the DNA damage response**. The evidence seems likely that diRNAs are induced from the vicinity of a DNA break. Furthermore, these diRNAs, although not required for phosphorylation of H2AX, they are required for the recruitment of MDC1 and 53BP1 to their modified histone binding partners. Whether the diRNAs remain at the site of chromatin and contribute to heterochromatinization of the region around the DSB is unknown. However, it is clear that the chromatin becomes more highly compacted, perhaps at later stages of DNA repair, and this promotes the process of DNA repair.

Several different types of non-coding RNAs have been implicated in the DDR. For example, many microRNAs (miRNAs) regulate genes that are involved in the DDR (reviewed in Wan et al., 2011; Han et al., 2012). However, these miRNAs are not necessarily encoded by genes in the vicinity of the DNA damage (**Figure 1**). Moreover, other non-coding RNAs are more directly involved in the DDR. Over 5% of the mammalian genome consists of short interspersed elements (SINEs), typified by the human *Alu* repeat. These are RNA polymerase III transcribed sequences whose expression is usually silenced. However, upon prolonged exposure to DNA damaging agents that predominantly cause DSBs, transcription from the human *Alu* elements and murine SINEs is strongly induced (Rudin and Thompson, 2001). The reason for the induction of the SINE/*Alu* non-coding transcription in response to DSBs is not clear, but it may be relevant that their silencing can be at least partially reversed by DNA demethylating agents in the absence of DNA damaging agents (Liu et al., 1994; Vorce et al., 1994). Pericentromeric DNA damage accumulation also leads to increased *Alu* transcript levels during adult human stem cell aging (Wang et al., 2011; this will be discussed more below). As such, it seems to be a reasonable prediction that the induction of SINE and *Alu* transcription in response to DSBs may be a consequence of decompaction of their normally repressive chromatin structure. In agreement with this, the association of histones with DNA is looser globally, as measured by their ability to be salt extracted, following treatment with DNA damaging agents (Xu et al., 2010). Similarly, transcription from the normally silenced yeast Ty retrotransposons is induced by exposure to a variety of DNA damaging agents including UV and γ-radiation (Morawetz, 1987; Bradshaw and McEntee, 1989; Morawetz and Hagen, 1990). Transcription of non-coding RNAs from Ty elements in response to DNA damage is unlikely to be beneficial to the host organism, given that this can lead to retrotransposition and insertion elsewhere into the genome, which itself is mutagenic. Insertion of new retrotransposon elements also increases the opportunity for unequal sister chromatid recombination.

It has been reported that new types of non-coding RNAs (rather than miRNAs or Alu elements) were induced by DNA damage, that are actually beneficial for DNA repair, in the filamentous fungus Neurospora crassa (Lee et al., 2009). However, this is not unique to Neurospora, as this has now been shown to be the case also in Arabidopsis and humans, as discussed below. In Neurospora, hydroxyurea and methyl methanesulfonate (agents that result in DNA breaks during replication) induce expression of a new class of small RNAs, about 20–21 nucleotides long, that originate mostly from the highly repetitive ribosomal DNA (rDNA) locus (Lee et al., 2009). These small RNAs originate from both strands of DNA in the region corresponding to the mature rRNAs but many also derive from the internal spacer regions of the rDNA. The remainder originates from genomic regions that encode transfer RNAs, other intergenic regions, and open reading frames. Production of these short RNAs requires RNA-dependent RNA polymerase, which can transcribe long RNAs that are 500 bp to 2 kb long (Lee et al., 2009). These long RNAs are then processed by Dicer to make the short RNAs, which bind to an active RNA-induced silencing complex (RISC) that includes Argonaute. Consistent with a role for these short RNAs in the DDR, Neurospora mutants lacking RNA-dependent RNA polymerase or Dicer are sensitive to DNA damaging agents. Because DNA damage is well known to result in a decrease in protein synthesis (Morley et al., 1998; Tee and Proud, 2000; Deng et al., 2002; Braunstein et al., 2009; Powley et al., 2009; Spriggs et al., 2010), Lee et al. (2009) hypothesized that production of the short DNA damage-induced RNAs from the rDNA may inhibit rRNA biogenesis and protein synthesis after DNA damage. Indeed, mutants defective in the synthesis of DNA damage-induced short RNAs failed to show a strong DNA damage-induced reduction in protein synthesis (Lee et al., 2009). Taken together, these results indicate that the production of small RNAs in Neurospora in response to DNA damage contributes to the DNA damage checkpoint by down-regulating protein synthesis.

One may ask: Why are small DNA damage-induced RNAs generated from the rDNA locus? The answer to this question may lie in the requirement for the single-strand DNA binding protein replication protein A (RPA) and the Neurospora counterpart of the Werner/Bloom syndrome helicase, QDE-1, for making small DNA damage-induced RNAs (Lee et al., 2010). A likely model is that the repetitive nature of the rDNA predisposes it to either DNA damage or the formation of aberrant structures upon replication stress, the repair or resolution of which requires unwinding of the DNA duplex by DNA helicases and entails processing of the rDNA into single-stranded DNA (ssDNA) coated by RPA. Consistent with this model, the RNA-dependent RNA polymerase uses ssDNA but not dsDNA as a template (Lee et al., 2010). Furthermore, RPA appears to recruit the RNA-dependent RNA polymerase to sites of ssDNA via their physical interaction. RPA also promotes the formation of dsRNA by the RNA-dependent RNA polymerase by disfavoring the formation of DNA/RNA hybrids (Lee et al., 2010). Taken together, the model for how small RNAs are generated from rDNA during replicational stress appears to be fairly sound. How these small DNA damage-induced RNAs result in downregulation of protein synthesis is less clear. The mechanism could be via small RNA-mediated degradation of the rRNAs or heterochromatinization of the rRNA locus, as both are mechanisms that are used in other scenarios in Neurospora (Dang et al., 2011).

A recent study showed that small non-coding RNAs can be induced around DSB sites in *Arabidopsis*. This work showed that small RNAs, referred to as diRNA (DSB-induced small RNAs), are induced in the vicinity of DSB sites, and revealed that these diRNAs promote repair of the DNA lesion (Wei et al., 2012). Generation of the diRNAs required the RNAi machinery such as Dicer and Argonaute 2 (AGO2). However, unlike the miRNAs that have also been implicated in the DDR (Hu and Gatti, 2011), diRNAs act in a manner distinct from translational inhibition through mRNA degradation. Following induction of a DSB using the endonuclease I-*Sce*I that cuts at a known location in the genome, all the diRNAs mapped specifically to the vicinity of the DSB site. The diRNAs mapped to both strands of the DNA, spanning a few kilobases on either side of the DSB. The generation of the diRNA depended on the PI3 kinase-related kinase ATR (Wei et al., 2012). While the phosphorylation of γH2A.X was not dependent on the induction of the diRNAs, the efficiency of DNA repair was greatly reduced when diRNA induction was blocked. The diRNAs bind to the AGO2 protein, which had previously been shown to be induced upon ionizing radiation (Culligan et al., 2006). Indeed ionizing radiation also resulted in the binding of AGO2 to diRNAs that were induced from 150 different genomic loci, suggesting that this is a general phenomenon in response to DNA lesions generated at many locations throughout the genome (Wei et al., 2012).

Mammalian cells also show a similar local induction of small RNAs upon DNA damage. Wei et al. (2012) demonstrated that diRNAs are induced around a DSB in human cells, with them being generated up to 6000 bp away from the DNA break. In human cells, knocking down Dicer or Ago2 significantly reduced the efficiency of DNA repair. Similarly, knocking down human Dicer or Drosha reduced the number of cells with 53BP1 localizing to repair foci, which is a protein involved in driving repair into the NHEJ pathway and its recruitment efficiency is affected by chromatin changes such as histone modifications including phosphorylation on histone H2AX S139 (γ-H2AX; Celeste et al., 2002) methylation on histone H4 lysine 20 (Botuyan et al., 2006), and histone H3 lysine 79 (Huyen et al., 2004; Wakeman et al., 2012), the histone acetyltransferase (HAT) activity of Tip60 (Murr et al., 2006) and recruitment of both MDC1 (Stewart et al., 2003) and RNF8 (Huen et al., 2007; Mailand et al., 2007). Knocking down human Dicer or Drosha also reduced the number of cells with foci of ATM autophosphorylation and the phosphorylated substrates of ATM, but did not reduce the number of cells with γH2A.X foci (Francia et al., 2012). As such, one can conclude that the DNA damage-induced RNAs act downstream of γH2A.X in the DDR. Interestingly, MDC1 recruitment to chromatin is via binding to γH2A.X (Stucki et al., 2005), yet knockdown of Dicer and Drosha also reduced MDC1 foci formation (Francia et al., 2012). This suggests that γH2A.X is not sufficient for MDC1 recruitment because diRNA are also required. Consistent with this idea, RNase A treatment of cells reduced the accumulation of MDC1 and 53BP1 in repair foci (Pryde et al., 2005; Francia et al., 2012), but had no effect on γH2A.X (Francia et al., 2012). MDC1 in turn is required for the amplification of the DDR signal (Lou et al., 2006), which would explain why ATM autophosphorylation and ATM-mediated phosphorylation of its substrates fails to occur upon the knockdown of Dicer and Drosha (Francia et al., 2012). One possible model that remains to be tested is that the diRNAs, or the process of their transcription *per se*, may help to generate an open chromatin structure that is permissive for the recognition of γH2A.X by its binding partners such as MDC1 (**Figure 1**).

## RNA-BINDING PROTEINS ARE RECRUITED TO DOUBLE-STRAND BREAK SITES

Perhaps related to the local transcriptional induction of small RNAs in response to DNA damage, several proteins that regulate RNA function have been shown to be transiently recruited to DNA damage sites. Adamson et al. (2012) found that the RNA-binding protein RBMX (RNA-binding motif protein encoded on the X chromosome) is recruited to DNA damage sites by genome-wide siRNA library screening. Furthermore, this appears to be important for DNA repair because silencing of RBMX leads to defects in HR-mediated DNA repair (Adamson et al., 2012). PPM1G, a phosphatase regulating splicing, is also recruited to DNA lesions (Beli et al., 2012). It has been reported that NONO, another RNA-binding protein, is recruited to a DSB site (Salton et al., 2010; Krietsch et al., 2012). Knockdown of NONO leads to a significant decrease in plasmid end joining and knockdown of NONO preferentially compromises NHEJ whereas HR is increased using *in vivo* NHEJ and HR reporter cell lines. Both RBMX and NONO are recruited to DNA lesions following laser-microirradiation in a poly-ADP-ribose polymerase (PARP)-dependent manner, where PARP mediates one of the earliest chromatin modifications in response to DNA damage (Adamson et al., 2012; Krietsch et al., 2012). Although the RNA targets at DSB sites that are bound by these factors are still unknown, these observations suggest possible active roles for RNA in DNA repair at DSB sites.

## TRANSCRIPTIONAL REPRESSION AROUND DOUBLE-STRAND BREAK SITES

The findings described above indicate that there is transcription in the immediate vicinity of DSBs in response to DNA damage. On the contrary, there is also evidence for transcriptional silencing induced in *cis* to a DSB, which may accompany, and facilitate, the later stages of DNA repair (**Figure 1**). Using a reporter that allows for visualization of the nascent transcript containing the MS2 RNA stem loop by binding to YFP-MS2 protein, silencing of transcription in *cis* to the DSB site has been observed (Shanbhag et al., 2010). However, DNA damage induced by ionizing radiation and laser-microirradiation does not cause global transcriptional silencing, indicating that the mechanism to induce transcriptional silencing only functions in the immediate proximity to the DSB (Shanbhag et al., 2010). Kruhlak et al. (2007) also reported that RNA polymerase I-mediated transcription of rDNA is inhibited in the vicinity of DSBs. Both forms of transcriptional silencing required ATM, suggesting that both pol I and pol II silencing may occur via a common mechanism around a DSB. Indeed, transcriptional arrest and dissociation of RNA polymerase II is induced around DSBs in active genes in a DNA-PK catalytic subunit-dependent manner (Pankotai et al., 2012). Consistent with the idea of transcriptional arrest, exclusion of the RNA processing factor THRAP3 (thyroid hormone receptor associated protein 3) from the vicinity of DSBs has also been shown, and this is dependent on the E3-ubiquitin ligases RNF8 and RNF168 (Shanbhag et al., 2010; Beli et al., 2012). Shanbhag et al. (2010) also observed ubiquitylated H2A around DSBs. H2A ubiquitylation has been strongly implicated in transcriptional silencing, suggesting that a repressive chromatin structure is formed around DSBs. DSB-induced chromatin condensation is likely to take place in *cis* not in *trans* since global chromatin decondensation is induced in response to DNA damage (Ziv et al., 2006).

Small non-coding RNAs are known to be involved in the formation of repressive heterochromatin structures over repetitive DNA elements, such as centromeric repeats and retrotransposons (Reyes-Turcu and Grewal, 2012). Although it has not yet been tested, it is possible that the diRNAs may function similarly to promote a more repressive chromatin environment around DSBs that may facilitate amplification of the DDR and DNA repair (**Figure 1**).

In previous sections, we discussed the possibility of chromatin decompaction and/or chromatin compaction by diRNA during the DDR. But what is the real function of diRNAs for regulation of chromatin structure around DSB sites? The chromatin structure around DSB site should be opened for protein recruitment (proximal to DSB site) and compacted for transcription repression (distal from DSB site). It is not clear which aspects of chromatin structure is regulated by diRNAs yet. There are several possibilities: (i) diRNAs could promote either open and closed chromatin structure around DSB sites. (ii) diRNAs could help make a boundary between closed and open chromatin structures around DSB sites. (iii) Compaction and/or decompaction of chromatin structures around DSBs by diRNAs may occur at different times during repair, or (iv) diRNAs may not directly regulate chromatin structure around DSBs but rather function through regulating the recruitment of DDR factors.

## HISTONE LOSS AND HISTONE GAIN DURING DNA DOUBLE-STRAND BREAK REPAIR

It is highly likely that the changes to the chromatin structure and transcriptional states described above that occur in response to DSBs are accompanied by the removal and replacement of histone proteins from the DNA. Histones H3/H4 are located at the center of the nucleosome, and their removal from DNA or their placement onto the DNA necessitates the removal or replacement of the entire histone octamer. This appears to occur, at least to some degree during DNA repair, because newly-expressed histone H3.1 is recruited to sites of UV-induced and laser-irradiation-induced DNA damage in mammalian cells (Polo et al., 2006). The histone chaperone CAF-1 (chromatin assembly factor 1) is required for this incorporation of H3.1 onto the newly-repaired DNA, and CAF-1 physically localizes to sites of DNA repair (Polo et al., 2006). The histone chaperone complex of HIRA/UBN1/CABIN1 is also recruited to microirradiation sites, however, their function in DNA repair is not clear yet (Adamson et al., 2012). Anti-silencing function 1 homolog A/B (ASF1A/B), a histone chaperone for H3/H4 dimers, also colocalizes with replication fork-associated repair foci. By immunoprecipitation assay, ASF1A/B are precipitated with the MMS22L (methyl methanesulfonate-sensitivity protein 22-like) DNA repair protein, and ASF1A and ASF1B colocalize with RPA foci under camptothecin (a topoisomerase inhibitor) or hydroxyurea (ribonucleotide reductase inhibitor) treatment, indicating that ASF1A and ASF1B are also recruited to replication fork-associated DNA damage lesions (Duro et al., 2010). However, the function of ASF1A/B in DNA repair is not clear, because HR efficiency or RAD51 foci formation is not affected by knockdown of ASF1A and/or ASF1B (Duro et al., 2010). Why are histone chaperones recruited to DNA damage site? One possibility is that histone chaperones are necessary for the restoration of chromatin structure in the wake of DNA repair. In budding yeast, *asf1* deletion mutants do not have a significant DNA repair deficiency (slightly delayed) but they do have reduced levels of histone H3 over the repaired DNA and defective recovery from checkpoint arrest after DNA repair (Chen et al., 2008). Similar results have been reported in a mammalian system. Depletion of ASF1A delays checkpoint recovery caused by UV irradiation (Battu et al., 2011).

Histones H2A/H2B occupy more peripheral positions on the nucleosome and they can be removed and replaced without the requirement for H3/H4 removal or replacement. Notably, PPM1G is recruited to DNA lesions in response to microirradiation (Beli et al., 2012), and it has been reported that PPM1G exerts H2A/H2B histone chaperone activity (Kimura et al., 2006). There is also evidence for the incorporation of variant versions of H2A in the vicinity of DNA breaks. For example, the DNA repair factor APLF functions as a histone chaperone, and is recruited to laser-microirradiation induced DSBs in a PARP-dependent manner (Mehrotra et al., 2011). One of two splice variants of macroH2A1, macroH2A1.1 (mH2A1.1) is recruited to DSBs, and its recruitment is dependent on its macrodomain [poly-ADP-ribose (PAR) binding domain] and the APLF histone chaperone (Timinszky et al., 2009; Mehrotra et al., 2011; Xu et al., 2012a). Chromatin structure around DSBs is rearranged by mH2A1.1 recruitment (Timinszky et al., 2009), even though it is not incorporated into nucleosome (Xu et al., 2012a). Depletion of mH2A1.1 leads to reduced 53BP1 foci and phospho-Chk2 levels but has no effect on γ-H2A.X (Xu et al., 2012a). Recently Xu et al. (2012b) reported that histone H2A.Z is rapidly deposited around DSBs after induction of DNA damage. This incorporation of H2A.Z around the DSB is required for H4 acetylation, RNF8-mediated ubiquitylation, and BRCA1-foci formation as well as Ku70/80 loading.

It is not clear yet how to connect histone chaperone activity and histone exchange with non-coding RNA transcription during DDR, especially around DNA damage sites. It is possible that the chromatin structure around the DNA damage site can affect non-coding RNA transcription and it should be further studied.

## NOVEL LINKS BETWEEN DNA DAMAGE, NON-CODING RNAs, AND CHROMATIN IN CELLULAR SENESCENCE AND AGING

Cellular senescence is mainly regulated by the p53/p21 and p16/pRB pathways. *lin*-*4* was the first identified miRNA which can regulate lifespan in *C. elegans* (Boehm and Slack, 2005) and a large number of other miRNAs have now been linked to the aging or senescence processes, for example, via regulating the p53/p21 and p16/pRB pathways. Some miRNAs that are expressed specifically during aging are secreted outside of cells, termed circulatory miRNAs, and are being studied as potential biomarkers of aging or for the diagnosis of age-related diseases. The influence of miRNAs in senescence and aging has been covered in detail in multiple recent excellent reviews (Smith-Vikos and Slack, 2012; Kato and Slack, 2013; Xu and Tahara, 2013) and will not be discussed here. Instead, we will discuss recent studies that have uncovered aging-dependent changes in the global chromatin structure, and how this relates to persistent activation of the DNA damage checkpoint and the concomitant induction of non-coding RNAs from repetitive elements such as the human *Alu* elements.

Telomere length is highly connected with the replicative lifespan of metazoan cells. Telomeres shorten with each successive round of DNA replication until one telomere reaches a threshold of “shortness” that emits a chronic DNA damage signal that causes the cell to cease dividing and enter senescence. Mechanistically, the short telomeres are seen as chronic DNA damage by the cell, resulting in the activation of the DDR, which causes a G1 cell cycle arrest (d’Adda di Fagagna et al., 2003). Telomere-mediated activation of the DDR appears to be intimately linked to the chromatin structure. Specifically, it was shown that chronic DNA damage from the processed telomeres also affects histone expression leading to their depletion and to the depletion of the central histone chaperones (O’Sullivan et al., 2010). The telomeric chromatin was progressively destabilized by this histone depletion, leading to a boost in telomere-associated DDR with each successive cell cycle (O’Sullivan et al., 2010). In a related study, the senescence-inducing chronic DNA damage signal caused by genotoxic stress was accompanied by reduced levels of the histone H2A variants in human fibroblasts (Lopez et al., 2012). By analogy, the level of total histone proteins decreases dramatically during replicative aging of budding yeast, but in this instance the trigger is not shortened telomeres but instead it appears to be due to a defect in protein synthesis during aging (Feser et al., 2010).

Perhaps related to the global relaxation of chromatin during aging, silenced regions of the genome become expressed. In yeast, replicative aging has long been known to be accompanied by the loss of transcriptional silencing (Smeal et al., 1996). This loss of silencing during yeast aging is likely due to both a reduction in histone protein levels during aging (Feser et al., 2010) and the reduced level of the Sir2 silencing protein (Dang et al., 2009).

Not all regions of the genome show relaxation of chromatin structure during aging, because senescent cells show heterochromatin foci called senescence-associated heterochromatin foci (SAHF; Zhang et al., 2005). The formation of SAHF is dependent on the histone chaperones ASF1A and HIRA and SAHF have a repressive histone variant, termed macroH2A (Zhang et al., 2005, 2007). It is not easy to distinguish when and where chromatin relaxation or condensation is occurred during cellular senescence. Besides, SAHF have not yet been observed in cells from old organisms although the levels of the essential factors to form SAHF (HIRA and macro H2A) are induced in cells from old organisms (Jeyapalan et al., 2007; Kreiling et al., 2011). Further studies are needed to determine how the chromatin structure changes over different regions of the genome during *in vivo* and *in vitro* aging (or senescence).

**FIGURE 2:**
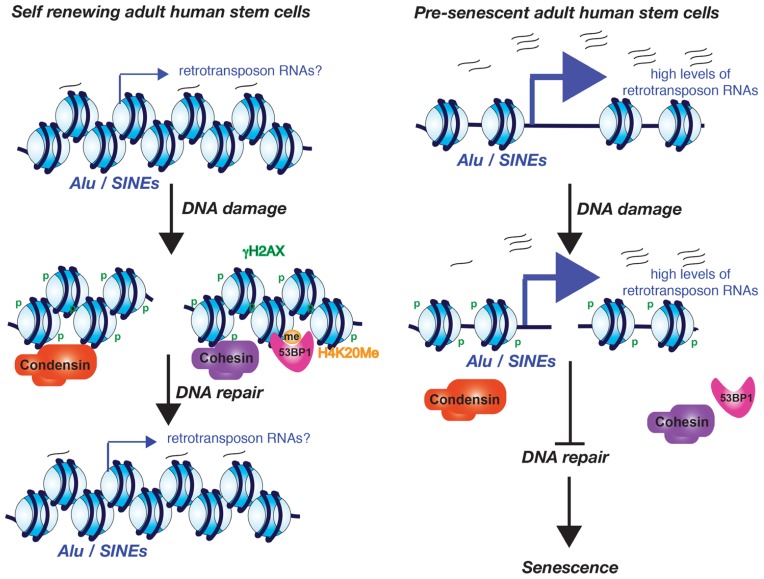
**Model for the role of excessive *Alu*/SINE RNA transcription in driving cells into senescence**. The model is based on the work from the Lunyak lab and is described in the text.

Increased transcription from the normally silenced *Alu* and SINE retrotransposons of human adult stem cells also occurs during aging (Wang et al., 2011). Whether this increased retrotransposon transcription is the consequence of the potential decay of the chromatin structure during aging is not yet known. Intriguingly, this *Alu*/SINE transcription appears to activate the DDR and subsequently causes senescence of adult human stem cells. This was all shown in an elegant study from the Lunyak lab, using *ex vivo* aging of human adipose derived mesenchymal stem cells where they noticed that the DNA damage foci that arose during aging and senescence localize to *Alu* retrotransposon/SINE elements and pericentromeric regions (Wang et al., 2011). *Alu*/SINE elements and pericentromeric chromatin are normally transcriptionally silenced. However during aging and senescence the *Alu*/SINEs were transcribed by RNA polymerase III (Wang et al., 2011). Furthermore *Alu*/SINE transcription may be required for the recruitment of 53BP1 to the DNA repair foci because a RNA pol III inhibitor blocked formation of 53BP1 foci (Wang et al., 2011). The age-dependent DNA damage foci at the pericentromeric DNA is accompanied by a localized failure to recruit cohesion and condensin 1 complexes during aging (Wang et al., 2011). Given that cohesion aids in DSB repair (Unal et al., 2004, 2007), failure to recruit cohesin during aging could explain the persistent DNA damage foci. If this is the case, the retrotransposon transcripts rather than retrotransposon transcription appear to be important for cohesion complex recruitment, given that knockdown of the *Alu*/SINE RNAs in senescent human adult stem cells caused loss of the persistent DNA damage foci and even restored their proliferative properties (Wang et al., 2011). This leads to a model where low levels of the retrotransposon transcripts are important for maintaining a repressive chromatin structure that allows the recruitment of cohesion and condensin I complexes that are important for the maintenance of pericentromeric integrity (**Figure 2**). However, in response to a potential decay of the chromatin structure during aging, excess retrotransposon transcription prevents recruitment of cohesion and condensin, causing persistent DNA damage checkpoint activation and senescence.

## REMAINING QUESTIONS

The recent papers discussed here have hinted at a complex interplay between the chromatin structure, transcription, DDR, and cellular senescence. However, many questions remain to be answered. These include: What is the function of non-coding RNAs in the DDR and cellular senescence? Are they involved in regulation of chromatin structures and/or recruitment of proteins to chromatin during the DDR and cellular senescence? What is the mechanism to induce non-coding RNAs during DDR and cellular senescence? Also, it will be interesting to determine whether the diRNAs that are transcribed from the vicinity of DSBs remain in the vicinity of the DSB. If so, this would be consistent with the diRNAs helping to physically establish a repressive chromatin structure? By contrast, if the diRNAs do not remain in the vicinity of the DSB, this would be suggestive that the act of transcription *per se* results in a more open chromatin structure, which would indirectly help recruit the DNA repair machinery such as 53BP1. Conversely, it is also a possibility that diRNA transcription around a DSB may be the consequence, not the cause, of local chromatin relaxation that occurs around the DSB. To determine if this is the case, it would be interesting to determine if the diRNAs are only produced over the domain that has lost histones or has H2AZ incorporation.

A better understanding of how diRNAs are induced in response to DNA damage may also be relevant for understanding how retrotransposon RNA transcription is induced during adult human stem cell aging. Is it a response to DNA breaks or a consequence of altered chromatin structure allowing access to the general transcription machinery? Do retrotransposon transcripts play a structural role in the maintenance of chromatin structure over these regions of the genome? Investigation of these and many other pertinent questions will doubtless provide mechanistic insight and more important discoveries.

## Conflict of Interest Statement

The authors declare that the research was conducted in the absence of any commercial or financial relationships that could be construed as a potential conflict of interest.
